# When Things Are Not as They Appear: Assessing the Adequacy of Cluster Randomization When Outcome Events Are Rare at Baseline

**DOI:** 10.1155/2012/806384

**Published:** 2012-05-08

**Authors:** Veronica Dinaj-Koci, Nanika Brathwaite, Lynette Deveaux, Sonya Lunn, Lesley Cottrell, Carole Harris, Bonita Stanton, Xiaoming Li, Sharon Marshall, Perry Gomez, Xinguang Chen

**Affiliations:** ^1^Carman and Ann Adams Department of Pediatrics, Wayne State University School of Medicine, Detroit, MI 48201, USA; ^2^The Bahamas Ministries of Health and Education, New Providence, Nassau, Bahamas; ^3^Department of Pediatrics, West Virginia University School of Medicine, Morgantown, WV 26506, USA; ^4^Health Research Center, West Virginia University, Morgantown, WV 26506, USA

## Abstract

The present study randomly assigned 15 Bahamian elementary schools to one of three intervention conditions. To assess the adequacy of cluster randomization, we examined two concerns identified by the local research team: inequality of gender distribution and environmental risk among groups. Baseline significant differences in risk and protective behaviors were minimal. There were significantly more males in the intervention group. Males had higher rates of risk behavior at all assessments. Poor school performance was also higher among the intervention condition and was significantly associated with increased rates of many but not all risk behaviors. Prior to adjusting for gender and school performance, several risk behaviors appeared to be higher after intervention among intervention youth. Adjusting for gender and school performance eradicated the group differences in risk behavior rates. Results demonstrate the importance of adequate randomization where outcomes of interest are rare events at baseline or differ by gender and there is an unequal gender distribution and the importance of the local research team's knowledge of potential inequalities in environmental risk (i.e., school performance). Not considering such individual differences could impact the integrity of trial outcomes.

## 1. Introduction

### 1.1. Dissemination of Effective Prevention Programs

With the growing awareness over the past decades that not all health promotion educational programs are equally effective, public health specialists and educators have urged the rigorous evaluation of prevention interventions and the subsequent utilization of only those found to be effective, for example, “evidence based” [1].

The utilization of evidence-based programs is becoming more achievable with the introduction of national programs such as the Center for Disease Control's (CDC) “Prevention Synthesis Project” and its “Dissemination of Effective Behavioral Interventions” (DEBI) program. The Prevention Synthesis Project has identified 69 prevention programs demonstrated through rigorous randomized, longitudinal evaluations to be effective in reducing one or more targeted risk behaviors [2, 3]. The DEBI program seeks to implement these interventions in a manner likely to retain their effectiveness, balancing the competing needs for fidelity to the original intervention with attention to specific local environmental conditions that require design alterations [4–6].

### 1.2. Integrating Effective Health Promotion Programs Into Schools

Worldwide, schools have been recognized as important vehicles for delivery of public health prevention programs [5, 7–10]. This recognition has served as the basis for school-based health prevention curricula across Africa [11–13], Asia [14, 15], and Latin America [16, 17] including family planning programs [18, 19], nutrition programs [20–22], and tobacco-prevention campaigns [23, 24].

As a practical matter, the efficacy trials of programs found to be effective have often been conducted in smaller settings than that of a school system [25]. Efforts to replicate the results of smaller efficacy trials in the context of large school systems may confront myriad implementation and evaluation issues [26] as well as limitations imposed by school regulations [8]. Attempts to minimize the impact of one design bias may unavoidably increase the risk of another bias. To avoid the potential of contamination of intervention conditions that may be more likely to occur when randomization is done at the level of the student or the class, researchers conducting school-based intervention trials commonly elected to randomize at the school level [8]. A review of smoking prevention programs found that 96% had been randomized at the level of the school [27]. However, allocation of participants by school may undermine the effectiveness of randomization in designing a trial, both because of the reduced number of units for randomization and because of the potentially greater heterogeneity between schools and/or greater homogeneity of the students or classes within schools. While ideally subjects assigned to various conditions should be comparable when the units for randomization are relatively homogenous and the process of randomization is independent of both the predictor and the outcome variables of a trial [28], researchers are nonetheless expected to assess if the randomly assigned groups are comparable with regard to key predictor or outcome variables. However, in situations in which the outcome or predictor variable(s) of interest is relatively uncommon and/or if there is a time lag between the intervention and the onset of the outcome of interest (such as early childhood exposures on the emergence of later life disease processes [29] or prevention interventions targeting risk or protective behaviors that are likely to arise months or years later [30]), it may be difficult to determine if the schools randomly allocated to the different conditions are equivalent until follow-up data becomes available. In situations in which an intervention has been delivered between baseline and followup, it may be impossible to determine if subsequent differences between the intervention and control groups resulted from the intervention or resulted from nonequivalence in the randomized units.

In a sexual risk prevention intervention specifically targeting preadolescents prior to sexual debut, there will be few sexually active youth at baseline in any of the participating schools and thus it will not be possible to determine if some schools are at higher risk than others are by comparing baseline rates of sexual initiation. Given the notion of cooccurrence of risk behaviors [31, 32]; comparing other risk behaviors may represent an alternative to assessing the effectiveness of the random allocation. However, in settings in which sexual onset occurs at a particularly young age, there may also be little involvement in other co-occurring risk behaviors, thus reducing the utility of this proxy approach. With limited rates of risk behaviors, researchers may be dependent on the knowledge of those familiar with local conditions, underscoring the importance of local partners to the integrity of a randomized trial. In such a situation, researchers may need to use other proxies for risk behavior. Among the possible candidates for proxies of sexual risk behavior are measures of academic achievement. Academic achievement is a plausible proxy for risk because a robust literature supports an inverse correlation between risk involvement and academic achievement [33, 34] suggesting that differences in levels of academic performance by school may compromise the validity of school-based randomization. Further, most schools will have some data regarding the academic achievement of their students compared to students in other schools. Consequently, among young children, academic achievement scores may provide an alternative index to assess the effectiveness of randomization independent of the outcome behaviors that were rare at baseline.

While ascertaining the relative proportion of males and females by intervention group is straightforward, controlling for differences in the distribution of behaviors that are attributable to gender but do not occur until after the intervention (e.g., are not apparent at baseline) also raises significant questions in data interpretation. When genders are equally distributed by intervention assignment, such potential confounding is not of concern. However, if randomization has not resulted in comparable gender distribution across the intervention conditions, then it will be important to assess the potential impact on intervention outcomes. While many risk behaviors may differ by gender and/or may vary by culture, earlier initiation of sex among males compared to females has been a consistent finding in most countries and cultures [35–39]. For example, among 33 nations from Africa, Asia, and Latin American included in a recent international survey, women initiated sex later than men in 31 nations; at a comparable age in one nation (Nigeria), and, at an earlier age in only one nation (Ghana) [39]. In the USA, analysis by gender among African American, Caucasian, and Asian youth found earlier onset among males, with a striking difference among African Americans; at age 13 years, 92% of females but only 72% of males were virgins and at age 14, 83% of females, and 58% of males remained virgins [36]. A survey conducted over a decade ago in Bahamian government high schools found that 57% of those 16 years and older were sexually experienced, including 70% of males and 41% of females [40].

In this paper we describe the methodological issues confronted in assessing the effectiveness of school-based randomization involving a relatively few number of schools (15) in a situation in which the outcome of interest (sexual risk) or proxies thereof (other risk behaviors) were rare events at the beginning of the trial given the young age of the subjects (grade 6 students). We present these issues in the context of evaluating the impact of an HIV prevention intervention delivered to preadolescents on subsequent risk and protective behaviors. Bahamian national members of our research team who were knowledgeable about the local conditions suspected that the randomization process had not resulted in comparable groups with regard to risk environment although baseline data indicated no differences in the key outcome variables (risk behaviors) because of the low frequency of these behaviors among the elementary school youth. We describe efforts to assess the adequacy of randomization using standard measures collected at baseline and followups (prevalence of sexual and other health risk behaviors and intentions and youth perceptions of risk behaviors among neighbors and family members) and, an index of academic performance (standardized school-based reading scores [33, 34]). We also address the difficulties in interpreting data when there are obvious gender imbalances across intervention groups and gender is significantly associated with one or more of the targeted outcomes (in this case, risk behaviors), but such outcomes (risk behaviors) do not appear until after baseline and administration of the intervention. 

## 2. Methods

### 2.1. The Setting: Preadolescent Sexual Risk Prevention in the Bahamas

The Bahamas has the second highest annual incidence of AIDS in the Caribbean; 2.2% of adults are infected with HIV. Heterosexual activity is the predominant mode of HIV transmission. Currently 57% of non-AIDS/HIV cases are among individuals 15 to 34 years old who represent <20% of the population [41]. Based on the high HIV rates and the high-risk sexual behavior among adolescents beginning in their early teen years, the Bahamian Ministries of Education and of Health decided to identify and adapt an HIV risk prevention intervention targeting preadolescents in Bahamian grade 6 classes, ultimately selecting the evidence-based program “Focus on Youth with Informed Parents and Children Together” (FOY+ImPACT) [42, 43] which they thought would be applicable to the Bahamas.

Therefore, the Ministries of Health and Education of the Bahamas invited the US developers to collaborate on the adaptation and evaluation of this pair of interventions for Bahamian grade six students and their parents. In contrast to the setting for the effectiveness trials in the USA that were community based (recreation centers and housing development, [42, 43]), The Bahamian government elected to conduct the trials within its government school system. The adapted program was entitled “Focus on Youth in The Caribbean” (FOYC) (a 10-session program plus two booster sessions) and a 1-hour parental monitoring intervention entitled “Caribbean Informed Parents and Children Together” (CImPACT)]. The US-Bahamas team evaluated the pair of risk reduction interventions through a randomized, controlled trial. The control condition for adolescents was a ten-session ecological intervention, titled “The Wondrous Wetlands” (WW) and the control condition for parents was a 20-minute career planning intervention, entitled “Goal for IT” (GFI).

Teachers and interested guidance counselors from the participating schools received a 5-day training workshop. The 10-session FOYC or WW curricula were delivered to students as part of the elementary school curriculum during class time. Parents received the appropriate intervention in groups on the weekend or evenings. Baseline and follow-up questionnaires (*The Bahamian Youth Health Risk Behavioral Inventory *(BYHRBI) [44], a cultural adaptation of the Youth Health Risk Behavior Inventory [45]), were administered to youth enrolled in the study to assess risk and protective knowledge, condom-use skills, perceptions, interventions, and self-reported behaviors.

We have described the trial in detail in prior publications [44, 46, 47]. Briefly, the trial was conducted among 1360 Bahamian sixth grade youth and 1175 of their parents. Youth and parents from 15 elementary schools located on the island of New Providence (from among the island total of 26 elementary schools) were randomly allocated at the school level to receive (a) FOYC+CImPACT (5 schools); (b) FOYC+GFI (5 schools); or (c) the WW+GFI (5 schools). Randomization was performed using the random digits method; in Year 1, nine schools were randomized to the three conditions (three schools per condition) and in Year 2, six schools were randomly added to each of the three conditions (two per condition). The analyses displayed hereon utilized data from one intervention group (FOYC+CImPACT) and the control group (WW+GFI), between which gender composition and several outcome variables were not comparable at baseline. As shown in Table 1 there were significantly more males in the FOYC+CImPACT group compared to the WW+GFI at baseline and at each follow-up assessment except the six-month followup. At all six waves the FOYC+CImPACT group was significantly older than the WW+GFI group. (Data from the FOYC+GFI group (5 schools) were analyzed and the findings are consistent with those presented here (data available upon request from the authors)).As reported in a prior publication [48], FOYC+CImPACT was found to increase condom use intentions, knowledge, condom-use skills, and perceptions at baseline and five follow-up assessments at 6, 12, 18, 24, and 36 months after intervention and condom-use behavior at 36 months followup. However, rates of sexual initiation were higher among FOYC+CImPACT youth compared to control youth at 36 months. 

### 2.2. Data Sources and Variables

Data used in this analysis were derived from two sources. Data used to assess *adolescent behaviors and perceptions* were derived from the assessment surveys (BYHRBI) we conducted during the randomized trial to evaluate the intervention trial. Data used to assess academic performance were derived from the annual English Grade Level Assessment Test (GLAT) as part of the Bahamas National Screening Programme [49]. More than 95% of elementary school students participate in the GLAT. The GLAT scores for 2005 are available in the public domain (URL: http://www.bahamaseducation.com/publication09.html).

**HIV/AIDS Knowledge**

Student knowledge of HIV/AIDS transmission and prevention was assessed through true-false questions. Six items assessing their transmission knowledge included questions about whether transmission could occur if an individual was kissing, touching, sharing needles, and so forth. Prevention knowledge was assessed by eight items on different ways of protecting against HIV infection. The scores were generated by the number of correct answers divided by the number of total of questions (scores ranging from 0 to 1).

**Condom-Use Skills**

The students were given 15 statements and were asked to choose the eight correct steps in using a condom. For each correct response, they were given a point. Final scores were reported as the mean number of correct responses (scores ranging from 0 to 1).

**Condom-Use Self-Efficacy**

Students' self-efficacy beliefs pertaining to condom usage were assessed through six questions assessing their self-perceived competency in hypothetical situations (5: strongly agree to 1: strongly disagree (Cronbach *α* = 0.87)).

**Response Efficacy**

Three items assessed the students' perceptions of the effectiveness of using condoms (5: strongly agree to 1: strongly disagree (Cronbach *α* = 0.71)).

**Adolescent Risk Behaviors and Perceptions**

Adolescents were assessed on 11 behavioral items covering three domains: substance use, delinquency, and sexual activity. Although FOYC+CImPACT did not specifically target all of the substance use and delinquent behaviors, there were discussions about the adverse effects of drugs and alcohol on decision-making. The decision-making model used throughout the 10 intervention sessions spoke to the importance of thoughtful decision making in all aspects of daily life. Thus it is plausible to presume that at least some of the risk behaviors might be modified by the decision-making intervention (and therefore might be seen as significant differences between the intervention conditions in the follow-up assessments), an effect seen in earlier evaluations of FOY [42]. Two items specifically targeted by the intervention asked whether the students had ever engaged in sexual intercourse and if so how consistent was their condom usage (“consistent condom use” defined as “always” using a condom). For each behavior, a 36-month prevalence rate was calculated to assess whether the students had ever performed any of the risk behaviors after entering the trial. In addition to individual level, these behavioral variables were also summarized by school to assess the overall risk involvement of students in each of the 15 participation schools. Analyses examining differences between groups were conducted overall and separately by gender. Questions were asked regarding intentions to engage in sexual risk (have sex in the next six months) and protective (use a condom in the next six months if you were to have sex) behaviors.

**Perceptions of Relative and Neighborhood Risk Behaviors**

The youths' perceptions of family and neighborhood behaviors were explored as an index of environmental risk. Youth reported how often they observed relatives and/or neighbors participating in substance-abuse-related activities. Items were coded as “sometimes or never” compared to “very often.” For each school, frequency of observing the behaviors “very often” was computed as a percentage score.

**Academic Achievement**

The 2005 English Grade Level Achievement Test (GLAT) scores by school for students in grade 6 were used as an assessment of the English language and writing performance. Passing rates (scored C or better) were computed for each of the 15 participation schools and were used in the analysis.

### 2.3. Statistical Analysis

 Assessing the comparability of the subjects randomized to the intervention (FOYC+CImPACT) and control (WW+GFI) conditions with regard to sexual risk could not be accomplished by comparing rates of sexual risk behaviors at baseline because the rates were so low. Therefore, we compared proxies of sexual risk including demographic variables associated with increased risk (age and male gender), nontargeted but potentially covarying risk behavior intentions to engage in sexual risk and/or use a condom were compared using logistic regression to compute odds ratios for categorical variables and ANOVA and effect sizes were calculated for continuous variables while correcting for baseline differences and age.

At baseline, there were differences in age among FOYC+CImPACT youth (mean = 10.5 and SD = 0.8) and WW+GFI youth (mean = 10.4, SD = 0.6, *P* < 0.05). In addition, there was a significant gender difference with 53% of the youth in FOYC+CImPACT being male compared to 46% of those in WW+CGI (*P* < 0.05) (see Table 1). To better assess the comparability of the two randomly assigned groups, the analyses were conducted for males and females, respectively.

To assess whether academic performances (as measured by the English GLAT scores) were distributed evenly across the schools randomized to the two intervention conditions; we summed the GLAT passing rates among the five schools in each of the three intervention conditions. We did not have GLAT scores for the individual students; rather we only had access to the average GLAT scores by school and without standard deviations. Therefore, we conducted one-sample *t*-tests to examine the difference between each school's GLAT score with the mean score of remaining 14 schools by using the GLAT of the case school as the test value (see “Potential Limitations” for discussion of the limitations of this approach).

We then examined the association of the English GLAT scores with the 11 variables assessing adolescent risk behaviors and six variables assessing perceived risk involvement by relatives and neighbors at the school level (*N* = 15) using correlation analysis. We reasoned that if there were a strong (e.g., coefficient *r* at *P* < 0.05) and directionally consistent (e.g., a consistent positive or a consistent negative) relationship between the GLAT score and a specific risk behavior over time, then the GLAT score at baseline could be viewed as a flag or indicator of significant propensity for the subsequent emergence of these risk behaviors. As such, if the GLAT scores were unevenly distributed across the randomly assigned intervention groups at baseline, this could indicate a lack of comparability of the randomly assigned groups.

 To adjust for differences in risk propensity that can be attributed to the English GLAT scores with regard to risk behaviors or perceptions, we computed the adjusted measures by intervention conditions using a multilevel approach with the measures of risk behaviors for individual students as level 1 factors and the 2005 GLAT passing rates for individual schools as a level 2 factor [50]. The English GLAT passing rates were included in the multilevel models for assessing risk behaviors at all follow-up assessments. The adjusted measures indicate what the levels of risk behaviors or perceptions would be if the passing rates of the English GLAT were comparable across the intervention conditions. These analyses were conducted among the cohort of all intervention youth and all control youth controlling for gender and among gender-specific subgroups.

Data for these analyses were manually entered. Data quality was examined before analyses were conducted. Detected errors were corrected against the original survey data. Statistical analysis was conducted using the commercial software SAS (SAS Institute Inc, Cary, NC). 

## 3. Results

As shown in Table 2, at baseline both intention to use a condom if having sex (a protective behavior) and intention to engage in sex in the next six months (a risk behavior) were significantly higher among FOYC+CImPACT youth compared to controls among the total sample and among the subsample of males. No significant differences in other sexual risk or protective perceptions, skills, or knowledge at baseline. Not shown in this table, GLAT scores were lower among FOYC+CImPACT schools (average 18.2 with three of the five schools having significantly lower mean scores of 11.2, 9.7 and 11.3 compared to the overall mean) compared to WW+GFI schools (average 24.2, with only one school having a significantly lower mean of 15.2 and one school a significantly higher mean of 31.7) (data available upon request).

Table 3 depicts the frequency distributions of risk behaviors by intervention group. Overall, at baseline there were no significant differences between FOYC+CImPACT and WW+GFI youth. Alcohol use was significantly higher among FOYC+CImPACT males compared to control males. There were no other significance differences by gender at baseline.

As shown in Table 2, postintervention, sexual initiation increased among both groups of youth, although the rate of initiation among all FOYC+CImPACT youth appeared to be faster than among WW+GFI youth; differences in sexual initiation between the two groups achieved statistical significance at 18 and 36 months. Sexual intentions were higher among FOYC+CImPACT in some waves. However, as noted above, given the significantly higher percentage of males in FOYC+CImPACT and the significantly higher rates of sexual initiation among males compared to females in both groups, the analyses were then conducted within gender subgroups. Among males only and among females only, there were no significant differences in either intentions or sexual initiation between FOYC+CImPACT and WW+GFI. 

Condom-use intentions, after controlling for baseline differences, were significantly higher at all followups except the six month follow-up among FOYC+CImPACT youth overall and among males; these differences were significantly higher among FOYC+CImPACT females at 18, 24, and 36 months. Condom-use intentions and behavior increased over time among both intervention and control youth and among males and females. Also apparent in Table 2, HIV knowledge, condom-use skills, and condom-use perceptions were significantly higher after intervention among FOYC+CImPACT youth. These differences were also apparent among the subset of males and the subset of females. The fact that differences were not present at baseline and that these postintervention effects did not differ by gender indicates an intervention rather than a sampling (randomization) effect.

Table 3 demonstrates substantial increases in six of the nine behaviors with age among both the FOYC+CImPACT and the WW+GFI youth; being suspended, smoking cigarettes and use of cocaine did not increase. Carrying a gun as a weapon and being involved in a physical fight was significantly higher among FOYC+CImPACT males, raising the possibility that the intervention may have in some way increased this risk behavior.

 Because it appeared that the significant increases in risk behaviors seen among the FOYC+CImPACT group were largely attributable to differences in gender composition of the two intervention groups, although sexual initiation remained somewhat higher overall even when controlling for gender, we explored the perception of our Bahamian local team members that the FOYC+CImPACT group was a higher risk group at baseline from an environmental effect (increased risk propensity). As noted above, the GLAT scores were somewhat lower among the FOYC+CImPACT schools compared to the WW+GFI schools. To explore the possibility that the GLAT scores might be a marker for future risk behaviors, in Table 4 we show the relationship between each of the 11 targeted risk behaviors and the GLAT score. With the exceptions of “suspended from school” and “consistent condom use” these relationships were all negative (e.g., the higher the GLAT score, the lower the frequency of risk behavior). Other than “suspended from school,” “alcohol use,” and “consistent condom use,” across 36 months of followup, these correlations achieved statistical significance for at least one of the follow-up periods. For “ever had sex,” these negative correlations were significant at all assessments. That is, regardless of intervention condition, youth in schools with lower GLAT scores were more likely to initiate sex. The negative correlations between the GLAT score and “sold or delivered drugs” and for carrying both guns and knives to “use as a weapon” were statistically significant. Table 4 also demonstrated the negative correlations between GLAT scores and perceptions of relatives and neighbor's involvement in four risk behaviors (using alcohol, marijuana and cocaine and selling drugs) at baseline, and each follow-up interval, with the exception of perceived frequency of relatives drinking alcohol. After baseline through 36 months followup, the majority of these correlations were significant.

 Therefore, in Table 5 we adjusted the rates for each of the eight behaviors which were significantly associated (in all cases negatively) at one or more assessment periods with the English GLAT scores and (gender for the overall group). The three behaviors without significant associations were adjusted without using GLAT scores. Following this adjustment “involved in a physical fight” (overall) and “carrying a gun as a weapon” remained higher (overall and among males) among FOYC+CImPACT youth. Sexual initiation, carrying a knife, and selling drugs overall were no longer significantly higher among FOYC+CImPACT youth. 

## 4. Discussion

The importance of using evidence-based approaches for disease prevention is well recognized; likewise, there is recognition of the need to reassess effectiveness of such interventions if they are significantly altered and/or applied in significantly different populations from that in which they were originally assessed [1]. School systems are often the site for “real-life” implementation of prevention programs targeting a broad audience [5, 10]. For a variety of logistic and methodological reasons, randomization will frequently be conducted at the level of the school and, as such, there may be relatively few units of randomization [8]. Because school populations are frequently geographic based, confounding due to possible homogeneity of underlying risk factors for the outcomes of interest may be problematic. If the outcomes and/or risk factors are relatively prevalent at baseline, adequacy of the randomization process could be assessed and/or baseline differences controlled for in the assessment of intervention impact. However, when the prevalence of outcomes of interest and/or known risk factors are low, baseline differences, which may impact outcomes, could be undetected. In such a case, utilization of available proxy measures would be of great importance in interpreting the results of such analyses. Likewise, if gender distribution is unequal across intervention groups and the outcomes of interest are of low frequency at baseline before the intervention, it may be difficult to disentangle the effects of gender and intervention assignment subsequently. Analysis by gender may be necessary to reveal actual (versus apparent) intervention impacts. In this study we show that both unequal gender distribution and inequality in baseline risk accounted for appearance of higher rates of sexual initiation and violence in the intervention group. By contrast, the higher rates of condom use, knowledge, and efficacy beliefs among FOYC+CImPACT youth appear to be true intervention effects.

In the present study, members of the local research team, familiar with the populations attending the elementary schools participating in the intervention evaluation, expressed concerns that the randomization process may have resulted in groups of unequal risk for the outcomes of interest (sexual risk behavior). However, sexual risk behavior and other potentially covarying risk behaviors were low at baseline and did not differ by intervention group at baseline. Overtime, with increased prevalence of risk behaviors, significant differences did emerge between the groups with regard to truancy, violence-related, and sexual risk behaviors. However, these differences emerged during the postintervention period.

Being aware of the correlation between academic achievement and risk behaviors in other studies, [33, 34] we explored the availability of such data at the level of the school, differences at the level of the schools, and association with risk behaviors. We found that a nationally administered academic examination (GLAT) was available for all schools, that the distribution of scores was not equivalent among schools in the three intervention groups and that the scores were highly (inversely) correlated with eight of the 11 risk behaviors, including one of the violence-related (gun-carrying) and sexual risk behaviors (initiation of sex) that did differ. Only three of the 11 risk behaviors we explored were not associated with the GLAT scores: having been suspended from school, failure to use a condom, and having drunk an alcoholic beverage. In the Bahamas the criteria for suspension varies greatly between schools as it is completely under the jurisdiction of the school principal or the viceprincipal (depending on the individual school). Therefore, behaviors, which might lead to suspension, differ greatly between schools and would vary over time as new principals are rotated through the school system. Thus, the practice does not reflect the local environment of the school or the neighborhood in which the school is located (personal communication, authors, L.C. Deveaux and S. Lunn). The relationship between failure to use a condom and other risk behaviors has been inconsistent across the age span and by culture [31, 51, 52]. Failure to use a condom is a complex behavior, first requiring that a youth engage in sexual intercourse (already a risk behavior) as well as negotiating several practical, personal, and social negotiations (obtaining a condom, feeling comfortable using a condom and convincing a partner to use a condom) [53]; arguably, it might be expected that such a complex behavior would not have a direct relationship with a general proxy of risk such as a lower GLAT score. Finally, unlike condomn use and school suspension, although there was an inverse correlation between GLAT scores and alcohol use, it did not reach statistical significance, perhaps reflecting the cultural acceptance of alcohol throughout the social stratum in the Bahamas.

 While it is possible that there is a direct impact of facility with language and/or academic achievement on risk behavior, it is perhaps more plausible that this score serves as an early identifier of risk behaviors before they appear. The 2009 Centers for Disease Control Youth Health Risk Behavior Survey confirms that students with higher academic achievement are significantly less likely to have carried a weapon, smoked cigarettes, consumed alcohol, or engaged in sex than their classmates with lower achievement (http://www.cdc.gov/HealthyYouth/health_and_academics/pdf/health_risk_behaviors.pdf).

Standardized assessments of academic progress are widely available and should be considered in situations such as that encountered here in which there is reason to believe that intervention groups may not be equivalent at baseline despite randomization. This study also speaks to the importance of the knowledge of local circumstances by the local research team. Their knowledge of the community environment should be seriously considered.

## Figures and Tables

**Table 1:** Age (SD) and gender distribution (%) at baseline and across intervention conditions among 933 youth participating in the FOYC+CImPACT versus WW+GFI trial (control).

		Total		Boys		Girls	
	Total	WW	FOYC	WW	FOYC	WW	FOYC
Sample							
Baseline	933(100.0)	497(100.0)	436(100.0)	228(45.9)	231(53.0)*	269(54.1)	205(47.0)*
6 months	884(94.7)	464(93.4)	420(96.3)	215(46.3)	221(52.6)	249(53.7)	199(47.4)
12 months	831(89.1)	432(86.9)	399(91.5)	202(46.8)	216(54.1)*	230(53.2)	183(45.7)*
18 months	785(84.1)	416(83.7)	369(84.6)	193(46.4)	202(54.7)*	223(53.6)	167(45.3)*
24 months	806(86.4)	417(83.9)	389(89.2)	190(45.6)	206(53.0)*	227(54.4)	183(47.0)*
36 months	752(80.6)	402(80.9)	350(80.3)	176(43.8)	182(52.0)*	226(56.2)	168(48.0)*

Age							
Baseline	10.5(0.7)	10.4(0.6)	10.5(0.8)*	10.4(0.7)	10.6(0.8)*	10.4(0.6)	10.43(0.8)
6 months	11.1(0.7)	11.0(0.7)	11.2(0.7)*	11.1(0.7)	11.2(0.7)*	11.0(0.7)	11.07(0.7)
12 months	11.6(0.7)	11.6(0.7)	11.7(0.8)*	11.6(0.7)	11.7(0.8)*	11.6(0.7)	11.62(0.8)
18 months	12.1(0.7)	12.1(0.7)	12.2(0.8)*	12.0(0.7)	12.3(0.8)**	12.1(0.6)	12.10(0.8)
24 months	12.6(0.7)	12.6(0.7)	12.7(0.8)*	12.7(0.7)	12.75(0.8)	12.5(0.6)	12.64(0.8)
36 months	13.6(0.7)	13.5(0.6)	13.7(0.8)*	13.6(0.7)	13.7(0.8)	13.5(0.6)	13.65(0.8)

*P < 0.05, **P < 0.01

**Table 2:** Sexual risk and protection intervention effect of youth participating in WW+GFI and FOYC+CImPACT overall and by gender controlling for age and baseline differences.

	Total				Boys				Girls			
	WW	FOYC	OR^¥^/ES	(95% CL)	WW	FOYC	OR/ ES	(95% CL)	WW	FOYC	OR/ES	(95% CL)
Sexual initiation %												
Baseline	4.13	5.33	1.26	(0.67, 2.38)	5.90	8.78	1.54	(0.73, 3.29)	2.68	1.42	0.49	(0.12, 1.97)
6 months	9.30	10.08	1.18	(0.64, 2.19)	16.34	15.99	0.96	(0.48, 1.89)	2.85	4.07	2.51	(0.45, 13.90)
12 months	14.71	17.75	1.36	(0.88, 2.10)	24.51	26.73	1.16	(0.70, 1.94)	5.75	7.74	1.59	(0.61, 4.14)
18 months	17.64	23.28*	1.54	(1.04, 2.93)	30.39	36.33	1.37	(0.86, 2.19)	6.17	8.61	1.63	(0.66, 4.06)
24 months	25.69	31.50	1.39	(0.99, 1.94)	41.06	45.14	1.20	(0.78, 1.84)	12.30	16.91	1.55	(0.83, 2.89)
36 months	33.14	43.73**	1.62	(1.19, 2.21)	51.36	60.69	1.48	(0.96, 2.28)	18.43	26.00	1.63	(0.97, 2.73)

Sex intention %												
Baseline	2.84	6.23*	2.28	(1.15, 4.52)	5.24	10.02	2.04	(0.96, 4.33)	0.80	1.97	2.45	(0.44, 13.79)
6 months	5.55	6.33	1.16	(0.64, 2.10)	10.52	9.92	0.93	(0.48, 1.82)	1.21	2.65	2.20	(0.52, 9.37)
12 months	5.13	10.30**	2.23	(1.25, 3.96)	9.26	16.75*	2.07	(1.09, 3.94)	1.31	2.89	2.16	(0.51, 9.23)
18 months	6.18	8.75	1.47	(0.82, 2.63)	10.61	14.10	1.42	(0.73, 2.77)	2.30	2.47	1.07	(0.28, 4.08)
24 months	6.08	9.08	1.59	(0.89, 2.81)	10.93	16.06	1.62	(0.85, 3.07)	1.84	1.67	0.90	(0.20, 4.13)
36 months	10.22	16.04*	1.73	(1.09, 2.73)	18.91	26.33	1.56	(0.92, 2.65)	2.94	5.40	1.85	(0.63, 5.46)

Condom use %												
Baseline	25.00	32.14	1.41	(0.08, 25.5)	37.10	30.64	0.76	(0.03, 19.37)	NE			
6 months	15.21	24.81	1.85	(0.60, 5.70)	19.55	28.78	1.67	(0.52, 5.33)	NE			
12 months	17.82	31.50	2.18	(0.92, 5.13)	20.68	35.23	2.13	(0.85, 5.35)	8.05	14.41	1.90	(0.12, 30.09)
18 months	18.80	24.34	1.39	(0.64, 3.06)	18.24	27.35	1.70	(0.71, 4.04)	20.08	7.60	0.27	(0.02, 3.94)
24 months	18.94	28.17	1.69	(0.89, 3.21)	19.67	26.50	1.48	(0.70, 3.15)	16.11	34.42	2.69	(0.73, 9.99)
36 months	32.00	44.50*	1.71	(1.05, 2.80)	33.26	52.86**	2.26	(1.26, 4.05)	29.78	22.07	0.65	(0.23, 1.83)

Condom Intention %												
Baseline	26.54	37.15**	1.64	(1.22, 2.20)	31.58	44.54*	1.74	(1.16, 2.63)	22.51	29.28	1.43	(0.92, 2.20)
6 months	40.89	44.81	1.18	(0.88, 1.59)	46.29	48.86	1.11	(0.73, 1.69)	36.07	40.97	1.24	(0.82, 1.90)
12 months	47.66	57.13*	1.49	(1.10, 2.01)	47.46	58.09*	1.58	(1.03, 2.42)	47.49	56.49	1.47	(0.96, 2.26)
18 months	39.70	60.28**	2.37	(1.74, 3.25)	43.74	57.85*	1.79	(1.15, 2.77)	36.03	63.48**	3.36	(2.12, 5.33)
24 months	53.56	69.41**	2.01	(1.47, 2.77)	52.33	64.14*	1.66	(1.06, 2.60)	54.23	75.23**	2.64	(1.66, 4.22)
36 months	68.51	77.94**	1.64	(1.15, 2.33)	65.35	76.44*	1.74	(1.05, 2.88)	70.73	79.66	1.64	(0.99, 2.73)

HIV knowledge												
Baseline	0.70	0.71	0.06	(0.05, 0.08)	0.68	0.70	0.17	(0.16, 0.19)	0.72	0.73	0.09	(0.07, 0.10)
6 months	0.74	0.80**	0.41	(0.40, 0.42)	0.71	0.79**	0.78	(0.77, 0.79)	0.77	0.82**	0.48	(0.47, 0.50)
12 months	0.57	0.59	0.11	(0.09, 0.13)	0.59	0.58	0.08	(0.06, 0.10)	0.56	0.60*	0.32	(0.30, 0.33)
18 months	0.56	0.63**	0.38	(0.36, 0.40)	0.59	0.64*	0.38	(0.37, 0.40)	0.54	0.62**	0.62	(0.60, 0.64)
24 months	0.55	0.62**	0.40	0.38, 0.42)	0.57	0.63**	0.49	(0.47, 0.51)	0.52	0.62**	0.80	(0.79, 0.82)
36 months	0.57	0.66**	0.46	(0.44, 0.48)	0.58	0.69**	0.82	(0.80, 0.84)	0.56	0.63**	0.50	(0.48, 0.52)

Condom skills												
Baseline	0.54	0.55	0.06	(0.04, 0.07)	0.57	0.56	0.08	(0.07, 0.10)	0.55	0.55	0.00	(−0.02, 0.02)
6 months	0.55	0.61**	0.34	(0.32, 0.35)	0.56	0.62**	0.48	(0.46, 0.50)	0.54	0.60**	0.47	(0.46, 0.49)
12 months	0.57	0.59	0.11	(0.09, 0.13)	0.59	0.58	0.08	(0.06, 0.10)	0.56	0.60*	0.32	(0.30, 0.33)
18 months	0.56	0.63**	0.38	(0.36, 0.40)	0.59	0.64*	0.38	(0.37, 0.40)	0.54	0.62**	0.62	(0.60, 0.64)
24 months	0.55	0.62**	0.40	0.38, 0.42)	0.57	0.63**	0.49	(0.47, 0.51)	0.52	0.62**	0.80	(0.79, 0.82)
36 months	0.57	0.66**	0.46	(0.44, 0.48)	0.58	0.69**	0.82	(0.80, 0.84)	0.56	0.63**	0.50	(0.48, 0.52)

Self-efficacy												
Baseline	2.37	2.26	0.09	(−0.20, 0.01)	2.55	2.40	0.18	(0.08, 0.29)	2.22	2.10	0.14	(0.03, 0.25)
6 months	2.81	3.20**	0.39	(0.29, 0.48)	2.93	3.34**	0.58	(0.48, 0.67)	2.71	3.05**	0.47	(0.38, 0.56)
12 months	2.81	3.16**	0.31	(0.21, 0.48)	2.99	3.37**	0.48	(0.37, 0.59)	2.65	2.92*	0.35	(0.24, 0.45)
18 months	3.00	3.51**	0.47	(0.36, 0.58)	3.17	3.74**	0.74	(0.63, 0.85)	2.84	3.25**	0.54	(0.43, 0.64)
24 months	3.12	3.60**	0.46	(0.36, 0.56)	3.35	3.83**	0.66	(0.55, 0.76)	2.92	3.34**	0.57	(0.46, 0.67)
36 months	3.49	3.91**	0.42	(0.32, 0.52)	3.82	4.21**	0.57	(0.47, 0.67)	3.23	3.58**	0.49	(0.39, 0.59)

Resp. efficacy												
Baseline	3.87	3.93	0.06	(−0.03, 0.15)	3.99	3.99	0.00	(−0.09, 0.09)	3.77	3.87	0.14	(0.05, 0.23)
6 months	3.98	4.24**	0.31	(0.23, 0.38)	4.05	4.30**	0.42	(0.34, 0.50)	3.91	4.18**	0.45	(0.37, 0.53)
12 months	4.00	4.26**	0.29	(0.20, 0.37)	4.06	4.32**	0.40	(0.32, 0.49)	3.94	4.20*	0.41	(0.32, 0.49)
18 months	4.08	4.39**	0.37	(0.28, 0.45)	4.27	4.37	0.17	(0.08, 0.25)	3.92	4.40**	0.81	(0.73, 0.89)
24 months	3.97	4.31**	0.41	(0.33, 0.49)	4.05	4.35**	0.51	(0.43, 0.59)	3.91	4.27**	0.61	(0.53, 0.69)
36 months	4.13	4.40**	0.33	(0.25, 0.41)	4.21	4.44**	0.41	(0.32, 0.49)	4.07	4.35**	0.48	(0.39, 0.56)

*P < 0.05, **P < 0.01; NE: nonestimable; ^¥^for categorical outcomes odds ratios were reported and effect sizes for continuous outcomes.

**Table 3:** Intervention effect of youth participating in WW+GFI and FOYC+CImPACT overall and by gender on nonsexual risk behaviors controlling for age and baseline differences.

	Total				Boys				Girls			
	WW	FOYC	OR^¥^	(95% CL)	WW	FOYC	OR	(95% CL)	WW	FOYC	OR	(95% CL)
Suspended from school												
Baseline	1.44	2.13	1.49	(0.50, 4.00)	1.24	2.84	2.28	(0.55, 9.36)	1.56	1.43	0.84	(0.18, 3.94)
6 months	0.92	1.48	1.63	(0.45, 5.88)	1.01	1.88	1.93	(0.34, 10.93)	0.84	1.04	1.15	(0.15, 8.67)
12 months	4.39	4.07	0.93	(0.44, 1.94)	5.73	5.90	1.03	(0.42, 2.52)	3.11	1.94	0.55	(0.13, 2.33)
18 months	8.95	6.49	0.69	(0.39, 1.24)	10.29	6.91	0.64	(0.29, 1.41)	7.88	5.99	0.73	(0.31, 1.72)
24 months	10.50	6.96	0.64	(0.36, 1.13)	11.15	6.84	0.59	(0.26, 1.33)	9.94	7.10	0.69	(0.31, 1.56)
36 months	16.88	12.81	0.72	(0.44, 1.17)	19.63	16.10	0.79	(0.41, 1.51)	14.80	9.42	0.59	(0.28, 1.25)

Carried a knife as a weapon												
Baseline	2.50	3.13	1.25	(0.57, 2.75)	4.90	5.63	1.16	(0.50, 2.66)	0.43	0.41	0.87	(0.04, 19.30)
6 months	2.59	4.39	1.85	(0.83, 4.12)	5.33	8.70	1.77	(0.78, 4.01)	0.00	0.00	NE	
12 months	2.26	5.40*	2.69	(1.20, 6.03)	4.12	8.77	2.31	(0.98, 5.49)	0.50	1.59	6.63	(0.36, 121.59)
18 months	4.59	7.95*	1.86	(1.01, 3.44)	9.22	14.20	1.63	(0.87, 3.18)	0.47	0.57	0.57	(0.02, 15.91)
24 months	4.59	8.08*	1.91	(1.04, 3.51)	8.83	13.50	1.66	(0.85, 3.22)	0.94	2.14	2.14	(0.37, 12.42)
36 months	10.75	13.78	1.34	(0.85, 2.10)	15.81	23.56	1.68	(0.97, 2.90)	6.65	3.56	0.52	(0.20, 1.37)

Carried a gun as a weapon												
Baseline	0.81	0.69	0.85	(0.19, 3.86)	0.87	1.31	1.52	(0.25, 9.30)	0.74	0.00	NE	
6 months	0.45	0.47	1.01	(0.14, 7.42)	0.48	0.92	1.92	(0.17, 21.70)	0.42	−0.02	NE	
12 months	0.47	0.75	1.58	(0.26, 9.64)	1.01	1.39	1.39	(0.23, 8.54)	0.00	0.00	NE	
18 months	0.00	1.93	NE		0.00	2.51	NE		0.00	1.22	NE	
24 months	0.73	0.77	1.06	(0.21, 5.32)	1.61	1.48	0.92	(0.19, 4.66)	0.00	0.00	NE	
36 months	1.08	4.81**	4.79	(1.58, 14.47)	1.87	7.64*	4.51	(1.26, 16.16)	0.42	1.82	4.17	(0.43, 40.81)

Involved in a physical fight												
Baseline	36.41	32.75	0.85	(0.65, 1.12)	44.49	36.88	0.73	(0.50, 1.06)	29.51	28.25	0.94	(0.63, 1.41)
6 months	28.87	24.83	0.81	(0.59, 1.10)	39.31	34.09	0.79	(0.53, 1.19)	20.14	15.78	0.74	(0.45, 1.21)
12 months	23.41	24.75	1.08	(0.78, 1.49)	30.70	34.55	1.20	(0.79, 1.82)	16.95	13.44	0.76	(0.44, 1.32)
18 months	31.89	32.87	1.05	(0.77, 1.42)	42.10	42.51	1.02	(0.67, 1.54)	22.97	21.56	0.91	(0.56, 1.50)
24 months	21.21	29.92**	1.60	(1.16, 2.22)	28.55	38.72*	1.60	(1.04, 2.46)	15.03	20.27	1.44	(0.86, 2.43)
36 months	26.23	25.75	0.98	(0.70, 1.34)	33.23	34.55	1.06	(0.68, 1.65)	20.79	16.27	0.74	(0.43, 1.25)

Smoked cigarettes												
Baseline	1.68	1.55	0.89	(0.31, 2.53)	2.82	1.58	0.51	(0.13, 1.95)	0.74	1.48	2.00	(0.33, 12.17)
6 months	2.36	2.95	1.25	(0.54, 2.89)	4.14	4.79	1.17	(0.46, 2.98)	0.77	1.06	1.18	(0.16, 8.76)
12 months	2.35	1.79	0.76	(0.28, 2.03)	3.98	2.38	0.59	(0.19, 1.85)	0.94	1.04	1.09	(0.14, 8.41)
18 months	1.96	3.54	1.83	(0.74, 4.51)	3.79	3.92	1.03	(0.36, 2.96)	0.44	3.00	6.82	(0.78, 59.34)
24 months	2.48	3.61	1.48	(0.64, 3.45)	3.28	4.90	1.52	(0.53, 4.41)	1.79	2.20	1.24	(0.30, 5.16)
36 months	1.62	3.06	1.94	(0.68, 5.58)	1.27	3.26	2.43	(0.45, 13.17)	2.00	2.70	1.39	(0.34, 5.69)

Drank alcohol												
Baseline	23.79	19.82	1.25	(0.57, 1.08)	29.17	20.99*	0.64	(0.42, 0.99)	19.17	18.54	0.96	(0.60, 1.53)
6 months	18.11	19.12	1.85	(0.75, 1.56)	21.53	24.28	1.20	(0.73, 1.96)	14.87	13.77	0.91	(0.52, 1.59)
12 months	14.27	19.80*	2.69	(1.04, 2.22)	20.43	22.74	1.16	(0.71, 1.90)	8.69	16.4*	2.10	(1.13, 3.89)
18 months	22.36	22.61	1.86	(0.71, 1.44)	26.25	24.55	0.90	(0.55, 1.46)	18.92	20.37	1.11	(0.65, 1.87)
24 months	18.19	24.07*	1.91	(1.02, 2.10)	20.61	27.61	1.52	(0.93, 2.50)	15.99	20.35	1.36	(0.80, 2.31)
36 months	26.46	34.05*	1.34	(1.06, 2.02)	25.97	31.65	1.35	(0.83, 2.18)	26.70	36.86*	1.63	(1.05, 2.54)

Used marijuana												
Baseline	0.22	1.15	5.50	(0.63, 47.77)	0.45	1.76	3.99	(0.44, 36.47)	0.01	0.48	NE	
6 months	0.66	0.97	1.47	(0.32, 6.68)	0.92	1.88	2.05	(0.37, 11.41)	0.41	−0.01	NE	
12 months	1.42	1.78	1.25	(0.41, 3.78)	2.53	3.25	1.29	(0.40, 4.18)	0.43	0.00	NE	
18 months	1.73	2.73	1.58	(0.59, 4.23)	3.67	4.02	1.10	(0.39, 3.12)	0.01	1.21	NE	
24 months	2.00	4.62*	2.37	(1.01, 5.56)	3.29	7.31	2.32	(0.87, 6.17)	0.90	1.64	1.82	(0.30, 11.23)
36 months	2.78	5.23	1.93	(0.90, 4.17)	5.77	7.23	1.27	(0.54, 3.00)	0.45	3.06	7.01	(0.80, 61.24)

Used cocaine												
Baseline	−0.02	0.72	NE		0.00	0.88	NE		−0.02	0.52	NE	
6 months	0.25	0.94	3.92	(0.42, 36.23)	0.53	1.81	3.63	(0.39, 33.54)	0.00	0.00	NE	
12 months	0.26	0.99	3.90	(0.43, 35.81)	0.53	0.44	0.80	(0.05, 13.94)	0.02	1.66	NE	
18 months	0.96	0.28	0.29	(0.03, 2.59)	1.57	0.50	0.32	(0.03, 3.15)	0.45	0.01	NE	
24 months	0.49	0.25	0.50	(0.04, 5.73)	0.56	0.74	0.81	(0.05, 13.85)	0.44	0.01	NE	
36 months	0.28	1.41	5.34	(0.61, 46.72)	0.64	2.17	3.52	(0.38, 32.83)	−0.02	0.62	NE	

Sold drugs												
Baseline	0.66	1.34	2.04	(0.50, 8.37)	0.95	2.14	2.31	(0.44, 12.32)	0.40	0.46	1.06	(0.06, 17.92)
6 months	1.15	1.16	0.93	(0.26, 3.36)	2.47	2.24	0.88	(0.24, 3.18)	0.00	0.00	0.00	
12 months	0.48	1.28	2.66	(0.51, 13.96)	1.02	2.37	2.31	(0.45, 12.49)	0.00	0.00	0.00	
18 months	1.44	1.97	1.37	(0.46, 4.13)	3.07	3.15	1.03	(0.32, 3.26)	0.00	0.61	NE	
24 months	1.11	3.52*	3.39	(1.09, 10.54)	1.79	5.82	3.57	(0.98, 13.04)	0.51	1.02	1.99	(0.17, 23.17)
36 months	2.86	3.12	1.08	(0.46, 2.57)	5.37	4.88	0.88	(0.33, 2.33)	0.86	1.28	1.38	(0.19, 10.05)

*P < 0.05, **P < 0.01 NE: nonestimable; ^¥^odds ratios.

**Table 4:** Standardized regression coefficients for GLAT scores^1^ predicting youth reported behavior over the past 6 months.

Youth self-reported:	Baseline	6 months	12 months	18 months	24 months	36 months	36 month prevalence
*Risk behaviors:*
Suspended from school	0.31	−0.31	0.12	0.28	0.16	−0.21	
Carried a knife to use as a weapon	−0.21	−0.32	−0.50	−0.54*	−0.42	−0.61*	
Carried a gun to use as a weapon	−0.07	−0.48	−0.25	0.19	−0.34	−0.59*	
Involved in a physical fight	−0.02	0.06	−0.43	−0.47	−0.64**	−0.52	
Smoked cigarettes	0.37	−0.53*	0.14	−0.34	0.06	−0.43	
Drank alcoholic beverages	−0.34	−0.13	−0.22	−0.36	−0.30	−0.01	
Used marijuana	−0.10	−0.09	−0.59*	−0.50	−0.35	0.01	
Tried any form of cocaine	0.05	0.23	.027	0.09	−0.54*	−0.63*	
Sold or delivered drugs	−0.04	−0.43	−0.18	−0.63	−0.64**	−.17	
Ever had sex	−0.70**	−0.64*	−0.54*	−0.65**	−0.73*	−.063*	
Consistent condom use	0.00	0.09	−0.46	0.06	−0.07	−0.16	

*Seen relative:*
Drink alcohol	0.10	−0.31	−0.27	0.33	−.07	0.00	−.25
Smoke marijuana	−0.50	−0.45	−0.42	−0.69*	−55*	−0.59*	−0.70**
Use cocaine	−0.45	−0.64**	−0.62*	−0.70**	−0.19	−0.46	−0.69**
Sell drugs	−0.51	−0.58*	−0.59*	−0.54*	−0.56	−0.39	−0.63*

*Seen neighbor:*
Drink alcohol	−0.40	−0.39	−0.62*	−.70**	−0.74**	−0.71**	−0.75**
Smoke marijuana	−0.46	−0.39	−0.53*	−0.65*	−0.74**	−0.74**	−0.73**
Use cocaine	−0.49	−0.54*	−0.67**	−0.76**	−0.66**	−0.58*	−0.80**
Sell drugs	−0.43	−0.70**	−0.53*	−0.70**	−0.67**	−0.75**	−0.76**

^1^Passing rates of the 2005 English Grade Level Achievement Test (GLAT) (scores of C or better) by school.

*P < 0.05, **P < 0.01.

**Table 5:** Rate of sexual and other risk behaviors by intervention condition after adjusting for GLAT score^1^.

	Total			Boys					Girls			
	WW	FOYC	OR	(95% CL)	WW	FOYC	OR	(95% CI)	WW	FOYC	OR	(95% CI)
Suspended from school												
Baseline	1.44	2.13	1.49	(0.50, 4.00)	1.24	2.84	2.28	(0.55, 9.36)	1.56	1.43	0.84	(0.18, 3.94)
6 months	0.92	1.48	1.63	(0.45, 5.88)	1.01	1.88	1.93	(0.34, 10.93)	0.84	1.04	1.15	(0.15, 8.67)
12 months	4.39	4.07	0.93	(0.44, 1.94)	5.73	5.90	1.03	(0.42, 2.52)	3.11	1.94	0.55	(0.13, 2.33)
18 months	8.95	6.49	0.69	(0.39, 1.24)	10.29	6.91	0.64	(0.29, 1.41)	7.88	5.99	0.73	(0.31, 1.72)
24 months	10.50	6.96	0.64	(0.36, 1.13)	11.15	6.84	0.59	(0.26, 1.33)	9.94	7.10	0.69	(0.31, 1.56)
36 months	16.88	12.81	0.72	(0.44, 1.17)	19.63	16.10	0.79	(0.41, 1.51)	14.80	9.42	0.59	(0.28, 1.25)

Drank alcohol												
Baseline	23.79	19.82	1.25	(0.57, 1.08)	29.17	20.99*	0.64	(0.42, 0.99)	19.17	18.54	0.96	(0.60, 1.53)
6 months	18.11	19.12	1.85	(0.75, 1.56)	21.53	24.28	1.20	(0.73, 1.96)	14.87	13.77	0.91	(0.52, 1.59)
12 months	14.27	19.80*	2.69	(1.04, 2.22)	20.43	22.74	1.16	(0.71, 1.90)	8.69	16.40*	2.10	(1.13, 3.89)
18 months	22.36	22.61	1.86	(0.71, 1.44)	26.25	24.55	0.90	(0.55, 1.46)	18.92	20.37	1.11	(0.65, 1.87)
24 months	18.19	24.07*	1.91	(1.02, 2.10)	20.61	27.61	1.52	(0.93, 2.50)	15.99	20.35	1.36	(0.80, 2.31)
36 months	26.46	34.05*	1.34	(1.06, 2.02)	25.97	31.65	1.35	(0.83, 2.18)	26.70	36.86*	1.63	(1.05, 2.54)

Carried a knife as a weapon												
Baseline	2.71	2.96	1.04	(0.39, 2.73)	4.97	5.55	1.14	(0.37, 3.51)	0.41	0.44	0.68	(0.01, 66.69)
6 months	2.60	4.26	2.00	(0.54, 7.14)	5.17	8.67	2.07	(0.46, 9.28)	0.00	0.00	NE	
12 months	2.91	4.67	1.61	(0.64, 4.02)	5.41	7.55	1.36	(0.50, 3.67)	0.45	1.65	8.46	(0.32, 221.62)
18 months	5.40	7.01	1.32	(0.65, 2.65)	10.09	13.37	1.40	(0.69, 2.86)	0.62	0.39	NE	
24 months	4.87	7.95	1.77	(0.92, 3.44)	8.92	13.42	1.63	(0.80, 3.30)	0.76	2.35	2.83	(0.38, 21.15)
36 months	12.08	13.06	1.06	(0.63, 1.76)	15.94	23.61	1.65	(0.76, 3.57)	7.46	2.74	0.41	(0.13, 1.26)

Carried a gun as a weapon												
Baseline	0.77	0.75	0.76	NE	0.81	1.41	0.03	NE	0.68	0.08	NE	
6 months	0.55	0.37	0.56	(0.05, 5.56)	0.56	0.84	1.46	(0.10, 20.83)	0.49	−0.10	NE	
12 months	0.64	0.56	0.91	(0.08, 6.25)	1.31	1.11	0.69	(0.08, 6.10)	0.00	0.00	NE	
18 months	−0.02	2.06	NE		0.28	2.25	NE		0.03	1.51	NE	
24 months	0.92	0.61	0.58	(0.09, 3.85)	1.89	1.23	0.59	(0.09, 3.85)	0.00	0.00	NE	
36 months	1.47	4.54*	3.80	(1.17, 12.31)	1.90	7.60*	4.41	(1.17, 16.68)	0.78	1.36	1.04	(0.04, 24.59)

Involved in a physical fight												
Baseline	36.92	32.34	0.99	(0.58, 1.12)	44.46	36.91	0.73	(0.49, 1.10)	29.64	27.74	0.90	(0.58, 1.39)
6 months	27.74	26.45	0.91	(0.23, 3.54)	35.27	36.82	1.06	(0.34, 3.32)	20.12	15.89	0.81	(0.44, 1.26)
12 months	24.15	23.66	1.00	(0.63, 1.49)	30.01	34.75	1.27	(0.73, 2.21)	17.97	12.14	0.62	(0.34, 1.13)
18 months	33.43	31.14	0.91	(0.65, 1.28)	43.19	41.29	0.92	(0.58, 1.44)	23.85	20.83	0.87	(0.51, 1.48)
24 months	22.10	29.33*	1.49	(1.05, 2.13)	29.75	37.62	1.44	(0.90, 2.30)	14.44	20.99	1.58	(0.91, 2.73)
36 months	27.85	24.65	0.84	(0.58, 1.21)	33.13	34.65	1.07	(0.66, 1.73)	21.98	14.66	0.59	(0.33, 1.07)

Smoked cigarettes												
Baseline	1.76	1.51	0.85	(0.28, 2.61)	2.83	1.57	0.52	(0.13, 2.14)	0.76	1.50	1.04	(0.07, 16.66)
6 months	2.72	2.62	0.92	(0.34, 2.34)	3.91	4.97	1.07	(0.39, 2.96)	1.12	0.58	NE	
12 months	2.52	1.57	0.61	(0.21, 1.83)	3.94	2.40	0.57	(0.16, 1.97)	1.13	0.80	0.78	(0.09, 6.80)
18 months	1.64	3.65	1.91	(0.33, 11.21)	3.73	3.78	1.04	(0.30, 3.58)	0.84	2.51	3.26	(0.30, 35.80)
24 months	2.60	3.52	1.38	(0.56, 3.42)	3.35	4.85	1.30	(0.38, 4.47)	1.67	2.35	1.37	(0.32, 5.91)
36 months	1.65	3.00	1.86	(0.60, 5.75)	1.13	3.40	2.76	(0.47, 16.28)	2.12	2.43	1.22	(0.17, 8.87)

Used marijuana												
Baseline	0.28	1.10	NE		0.44	1.77	NE		0.06	0.42	NE	
6 months	0.47	1.13	3.04	(0.20, 45.63)	0.71	2.08	2.81	(0.26, 30.89)	3.13	0.10	NE	
12 months	1.63	1.52	0.76	(0.17, 3.43)	3.12	2.70	0.77	(0.20, 3.00)	0.35	0.45	NE	
18 months	1.95	2.47	1.24	(0.42, 3.68)	3.71	3.97	1.07	(0.34, 3.35)	0.20	1.24	NE	
24 months	2.10	2.54	2.69	(0.52, 13.93)	3.62	6.89	1.89	(0.57, 6.28)	0.71	1.89	2.85	(0.21, 39.15)
36 months	2.78	5.43	1.83	(0.80, 4.18)	5.34	7.65	1.44	(0.58, 3.60)	0.58	2.88	5.76	(0.60, 55.65)

Used cocaine												
Baseline	0.27	0.94	NE		−0.07	0.95	NE		−0.07	0.60	NE	
6 months	0.32	0.97	3.60	(0.36, 35.82)	0.50	1.83	3.60	(0.36, 35.94)	0.00	0.00	NE	
12 months	0.33	0.96	3.08	(0.14, 65.86)	0.73	0.25	NE		0.04	1.72	NE	
18 months	1.00	0.23	0.23	(0.02, 2.55)	1.55	0.49	0.28	(0.02, 4.66)	4.39	0.00	NE	
24 months	0.45	0.30	0.64	(0.05, 7.85)	0.30	0.70	1.44	(0.08, 26.20)	0.54	−0.12	NE	
36 months	0.43	1.30	3.74	(0.37, 37.86)	0.74	2.07	3.08	(0.29, 32.56)	0.09	0.48	NE	

Sold drugs												
Baseline	0.60	1.45	2.36	(0.55, 10.18)	0.73	2.44	3.09	(0.56, 17.09)	0.43	42.00	0.74	(0.02, 23.60)
6 months	1.30	1.04	0.70	(0.10, 5.01)	2.71	2.00	0.70	(0.17, 2.89)	0.00	0.00	NE	
12 months	0.50	1.22	2.57	(0.14, 47.32)	0.98	2.36	2.55	(0.14, 46.72)	0.00	0.00	NE	
18 months	1.80	1.55	0.77	(0.21, 2.85)	3.57	2.65	0.67	(0.17, 2.61)	0.09	0.48	NE	
24 months	1.38	3.28	2.52	(0.75, 8.48)	2.22	5.30	2.82	(0.72, 11.14)	0.58	1.02	1.50	(0.03, 70.25)
36 months	2.69	3.49	1.12	(0.45, 2.82)	4.05	6.07	1.04	(0.34, 3.18)	0.89	1.23	1.30	(0.16, 10.33)

Sexual initiation												
Baseline	4.67	4.82	2.17	NE	6.38	8.39	2.85	NE	2.99	1.01	NE	
6 months	9.46	9.90	1.07	(0.36, 3.15)	16.38	15.75	0.95	(0.11, 8.11)	2.85	4.07	2.64	(0.44, 15.84)
12 months	14.43	17.81	1.51	(0.45, 5.10)	23.33	27.85	1.44	(0.49, 4.23)	5.49	8.09	1.97	(0.74, 5.24)
18 months	18.03	22.45	1.45	(0.81, 2.59)	29.93	36.48	1.20	(0.62, 2.32)	6.11	8.70	1.81	NE
24 months	27.66	29.88	1.16	(0.79, 1.69)	43.01	43.34	1.05	(0.67, 1.63)	12.32	17.09	1.81	(0.96, 3.42)
36 months	35.10	43.08	1.39	(0.97, 1.86)	52.72	59.44	1.19	(0.79, 1.81)	18.02	26.55	1.64	(0.93, 2.89)

Condom use												
Baseline	25.00	32.14	1.41	(0.08, 25.5)	37.10	30.64	0.76	(0.03, 19.37)	NE			
6 months	15.21	24.81	1.85	(0.60, 5.70)	19.55	28.78	1.67	(0.52, 5.33)	NE			
12 months	17.82	31.50	2.18	(0.92, 5.13)	20.68	35.23	2.13	(0.85, 5.35)	8.05	14.41	1.90	(0.12, 30.09)
18 months	18.80	24.34	1.39	(0.64, 3.06)	18.24	27.35	1.70	(0.71, 4.04)	20.08	7.60	0.27	(0.02, 3.94)
24 months	18.94	28.17	1.69	(0.89, 3.21)	19.67	26.50	1.48	(0.70, 3.15)	16.11	34.42	2.69	(0.73, 9.99)
36 months	32.00	44.50*	1.71	(1.05, 2.80)	33.26	52.86**	2.26	(1.26, 4.05)	29.78	22.07	0.65	(0.23, 1.83)

*P < 0.05, **P < 0.01; NE: nonestimable.

^1^Passing rates of the 2005 English Grade Level Achievement Test (GLAT) (scores of C or better) by school.
